# Isolation, Identification, and Control of Pathogenic Endophytic Fungi in *Nymphaea candida* Presl Tissue Culture

**DOI:** 10.3390/microorganisms13051103

**Published:** 2025-05-10

**Authors:** Yuwei Xing, Cong Liu, Xumeng Cui, Haonan Lv, Jun Wang

**Affiliations:** Colleges of Marine Life Sciences, Ocean University of China, 5 Yushan Road, Qingdao 266003, China; 15335362168@163.com (Y.X.); liucong6899@163.com (C.L.); simone_c@163.com (X.C.); lhn113@163.com (H.L.)

**Keywords:** *Nymphaea candida* Presl, tissue culture, pathogenic endophytic fungi, microbial pollution prevention and control, endangered plant breeding

## Abstract

*Nymphaea candida* Presl is a rare hardy water lily at risk of extinction and has been included on the ‘Red List’ of threatened species of the International Union for the Conservation of Nature. To protect germplasm resources and propagate *N. candida* seedlings, this study conducted tissue culture and found that pathogenic endophytic fungal infection was the main reason for failure of tissue culture. Compared with the stems and leaves, the roots of *N. candida* had the highest rates of fungal infection during tissue culture. Subsequently, three isolated endophytic fungi, *Fusarium oxysporum*, *Phytopythium helicoides*, and *Alternaria* sp., showed the highest frequency of occurrence in tissue culture. Furthermore, an antifungal formulation comprising 0.1 μg/mL pyrimidin suspension, 1 μg/mL mancozeb wettable powder, and 1 μg/mL carbendazim was constructed and could reduce the infection rates of root and stem tissues to 7.5% and 0%, respectively. Finally, the usefulness of this antifungal formulation for inhibition of endophytic fungi in tissue culture of *N. candida* was validated. This study not only provides important technical support for mass production of seedlings of *N. candida*, but also provides a scientific reference for the protection of endangered aquatic plant species.

## 1. Introduction

There are more than 50 kinds of water lilies in the world [1], and they are divided into tropical and hardy water lilies according to their temperature demand for survival [2]. *Nymphaea candida* Presl, a hardy water lily, is mainly distributed in Siberia, Poland, and Mongolia. In China, *N. candida* is only distributed in Bosten Lake, Xinjiang Uygur Autonomous Region [3,4,5]. Because the petals of *N. candida* contain abundant flavonoids, saponins, and phenols, which have antibacterial, anti-inflammatory, and anti-oxidation effects, *N. candida* have been widely used in anti-virus, anti-pyretic, and anti-alcoholism medicines [4]. As a floating-leaved plant, *N. candida* not only effectively absorbs nitrogen and phosphorus pollutants in the water, but also has a good removal effect on heavy metals (lead and mercury) in sediments [5]. In Bosten Lake, *N. candida* provides natural refuges and nurseries for a variety of waterfowl species [6]. However, the distribution area of *N. candida* is decreasing constantly owing to habitat degradation, agricultural activities, and water quality deterioration [4]. To prevent the extinction of this endangered species, *N. candida* has been included on the ‘Red List’ of threatened species of the International Union for the Conversation of Nature (IUCN) and the List of National Key Protected Wild Plants in China. It is urgent to strengthen the protection of the germplasm resources and the propagation of *N. candida*.

The germination and growth of *N. candida* is easily affected by diseases, and moreover most of the seeds are eaten by birds, resulting in a very low natural reproduction rate and population growth rate [7]. It is necessary to develop artificial breeding and seedling culture techniques for *N. candida*. Plant tissue culture based on plant cell totipotency could use isolated organs and tissues to induce callus, roots, and adventitious buds under aseptic conditions and finally regenerate complete plants [8]. Tissue culture has become an important tool for plant rapid propagation for many terrestrial and aquatic plants, including seaweed, *Indian lotus*, *Spirogyra*, and *Hygrophila polysperma* [9,10,11,12,13]. Moreover, plant tissue culture technology has been successfully used for the conservation and breeding of endangered species [14]. Through tissue culture, a large quantity of seedlings of critically endangered species, including *Cirsium hillii*, *Anoectochilus elatu Lindley*, and *Rubus humulifolius*, were obtained, and their seedlings were successfully introduced into the Bruce Peninsula National Park (Canada), the National Orchid Garden in Yerkod, Tamil Nadu (India), and the Botanical Garden of the University of Oulu (Finland), respectively [14,15,16]. We considered that tissue culture technology might be an effective approach for the reproduction of wild *N. candida*.

The success of plant tissue culture is closely related to the prevention and control of microbial contamination [17]. The incomplete sterilization of plant explants usually leads to microbial contamination of the medium, which contains many nutrients and vitamins [18]. The growth of microorganisms would make explants fail to form a callus [19]. Epiphytic microorganisms can be readily killed by disinfecting the surface of explants, and thus the prevention of endogenous microbial contamination became crucial [18]. Antibiotics, including ampicillin and iminoctadine triacetate, are usually used to prevent endophytic bacterial contamination [20]. By contrast, endophytic fungal contamination is more difficult to prevent and usually causes callus growth retardation and even explant death [21]. For example, proliferation of the endophytic fungi *Cladosporium* and *Plectosphaerella* resulted in a failure rate of 40–60% in tissue culture of *Hygrophila polysperma* [19]. However, the use of antibiotics for the prevention of endophytic fungi including *Peniophora* sp., *Plectosphaerella oligotrophic*, *Cladosporium crousii*, and *Dark septate endophytes* usually inhibited the growth of explants and seriously reduced the success rate of plant tissue culture [22,23,24]. It is necessary to optimize the dosage and combination of antimicrobial drugs. We speculated that the success rate of tissue culture of *N. candida* could be significantly improved by choosing appropriate antifungal drugs to prevent endophytic fungal infections.

In this study, the root, stem, leaf, flower, and bud tissues of *N. candida* were used for tissue culture, and the infected fungi were isolated and identified. Meanwhile, high-throughput sequencing technology was used to analyze the diversity and community structure of endophytic fungi in the roots, stems, and leaves of *N. candida*. Subsequently, five drugs were selected to inhibit three main pathogenic fungi in tissue culture, and the doses of the drugs were optimized. Finally, the optimal drug combination was used for tissue culture of *N*. *candida* to evaluate its reliability. This study provides a scientific basis for reducing pollution of endophytic fungi and improving the success rate of tissue culture for mass production of aquatic plant species.

## 2. Materials and Methods

### 2.1. Biomaterials and Reagents

*N. candida* was collected from Bosten Lake (86°40′00″–87°25′00″ E, 41°56′00″–42°14′00″ N) in Bohu County, Bayingolin Mongol Autonomous Prefecture, Xinjiang. Potato Dextrose Agar Medium (PDA) and Murashige and Skoog media (MS) were purchased from Aladdin Biochemical Technology Co., Ltd. (Shanghai, China). Thiabendazole (TDZ) and 1-naphthaleneacetic acid (NAA) were purchased from Bokang Biotechnology Co., Ltd. (Qingdao, China). Carbendazim, sodium dichloroisocyanurate, chlorothalonil wettable powder, pyrimidin suspension, and mancozeb wettable powder were purchased from Sinopharm Chemical Reagent Co., Ltd. (Shanghai, China).

### 2.2. Tissue Culture of N. candida and Fungal Contamination

From August to September 2024, this experiment was conducted at the College of Marine Life Sciences, Ocean University of China, Qingdao City, Shandong Province, China (120°34′36″ E, 36°07′25″ N). Roots, stems, leaves, flowers, and buds (5–6 cm samples) were cut from healthy *N. candida* plants and rinsed with running water for 5–10 min to remove the surface sludge. These tissues were cut into small pieces of approximately 1 cm, immersed in 75% ethanol for 10 s, rinsed with sterile water, immersed in 0.1% mercuric chloride solution for 12 min, and then rinsed with sterile water five times to complete surface disinfection treatment. The MS basal medium was supplemented with 3.00 mg/L TDZ and 0.20 mg/L NAA, and the pH was adjusted to 5.8 ± 0.1 with 1 mol/L HCl. The medium was added to a 240 mL culture flask for autoclave sterilization (121 °C, 15 min). After cooling and solidification, 50 mL sterile distilled water was added to prepare a double-layer medium. The surface-disinfected tissues were inoculated into the double-layer medium and cultured at 28 °C in the dark. Growth of the explants was observed every 3 days until callus differentiation, and the infection of each tissue was recorded and photographed [15].

### 2.3. DNA Extraction and Amplicon Determination of Endophytic Fungi in Roots, Stems, and Leaves of N. candida

The root, stem, and leaf tissues of *N. candida* were separately cut into small pieces of approximately 0.5 cm, completely surface-disinfected, and then ground to powder in liquid nitrogen. Total DNA was extracted from the samples using a DNeasy Power Soil kit (Qiagen, Hilden, Germany) according to the instructions. The quality and concentration of the DNA were determined by 1% agarose gel electrophoresis and spectrophotometry, respectively. Fungal universal primers for ITS1F (5′-CTTGGTCATTTAGAGGAAGTAA-3′)/ITS2 (5′-GCTGCGTTCTTCATCGATGC-3′) were used to amplify the full-length ITS rDNA genes of endophytic fungi, and the amplified products were sent to Sangon Biotech Co., Ltd. (Shanghai, China) for sequencing [25].

### 2.4. Isolation and Purification of Endophytic Fungi

The endophytic fungi detected during tissue culture were isolated according to the methods of Li et al. [19]. Firstly, a medium sample with a diameter of approximately 5 mm containing different phenotypic hyphae was cut out using an inoculation needle and placed in the center of a new PDA medium plate. The culture was repeated three to five times until the strains were completely purified. The purified strains were then inoculated onto PDA inclined planes by the streaking method. When the inclined planes were covered with mycelia, they were placed at 4 °C for temporary storage.

### 2.5. Identification of Endophytic Fungi and Construction of a Phylogenetic Tree

The purified strains were streaked onto PDA plates and cultured at 28 °C for 48–96 h to observe and photograph the morphology of endophytic fungi [26]. Subsequently, the purified endophytic fungal hyphae were ground with sterile glass beads, and the genomic DNA was extracted using Invitrogen Genomic DNA Extraction Kits (Invitrogen Corporation Shanghai Representative Office, Shanghai, China). NS1 (5′-GTAGTCATATGCTTGTCTC-3′)/NS6 (5′-GCATCACAGACCTGTTATTGCCTC-3′) primers were used for PCR amplification, and PCR products were analyzed by 1% agarose gel electrophoresis. The target band was recovered and purified using a purification kit, and the purified PCR products were sent for Sanger sequencing by Sangon Biotech Co., Ltd. The obtained sequences were subjected to blast sequence homology comparison (https://blast.ncbi.nlm.nih.gov/Blast.cgi, accessed on 7 May 2025) with other fungal sequences in the National Center for Biotechnology Information (NCBI) database with a similarity greater than 99% [27]. Multiple sequence alignment analysis was performed on the purified strains and related strains using MEGA 7.0.14, and the phylogenetic trees were constructed using the Neighbor-Joining method [28]. The raw sequencing data have been deposited in the NCBI Sequence Read Archive under accession number PRJNA1248482.

### 2.6. Inhibitory Effect of Different Drugs on Pathogenic Endophytic Fungi

Five antifungal drugs, including sodium dichloroisocyanurate, carbendazim, chlorothalonil wettable powder, pyrimidin suspension, and mancozeb wettable powder, were selected to inhibit the three isolated pathogenic fungi: *Alternaria* sp., *Phytopythium helicoides*, and *Fusarium venenatum* (Table S1). A total of 20 groups for five drugs with different concentrations were set up. Meanwhile, a control group (PDA plate without any drug) completed the process. First, the PDA medium was autoclaved (121 °C, 0.105 MPa, 15 min) and cooled to 40–50 °C. Then, the five antifungal drugs at concentrations of 0.01, 0.1, 1, and 10 μg/mL were separately added to the PDA medium to prepare the plates. Subsequently, *A.* sp., *P. helicoides*, and *F. venenatum* were inoculated onto the plates (n = 6) for each group and placed in a constant-temperature incubator (Boxun Industrial Co., Ltd., Shanghai, China) at 28 °C for 3 days. The colony morphology was observed, and the colony diameter of each group was measured by the cross-crossing method. The growth inhibition rates of different concentrations of the five tested drugs against the three pathogenic fungi was calculated according to the method of Sheikholeslami et al. [15] to determine the minimum inhibitory concentration of each drug. Based on the results, the optimal drug dosages were selected for the following experiments.

### 2.7. Application of Antifungal Drugs in Tissue Culture

MS medium (50 mL) with 3.00 mg/L TDZ and 0.20 mg/L NAA was added to 100 mL plastic culture containers, and the pH was adjusted to 5.8 ± 0.1 with 1 mol/L HCl prior to autoclave sterilization. When the medium was cooled to 40–50 °C, the combined antifungal drugs were added into the medium and cooled at room temperature until it was solidified. Then, 50 mL sterile distilled water was added to prepare a double-layer medium, and surface-sterilized explants were inoculated onto the solid medium. The infection rate and growth state of the explants in the tissue culture bottle were observed and recorded during tissue culture.

### 2.8. Statistical Analysis of the Data

Data processing and statistical analysis were performed using IBM SPSS Statistics 22 (version 27.0). The significance of the differences between groups was analyzed using one-way ANOVA after data were confirmed to fulfill the criteria of normal distribution and equal variance (ANOVA, LSD, *p* < 0.05). The alpha diversity index was calculated by Mothur (version 1.35.1), and ANOSIM was used to evaluate differences in endophytic fungal community groups in root, stem, and leaf tissues. The Bray–Curtis distance matrix was calculated, and the beta diversity of the community was analyzed using non-metric multidimensional scaling (NMDS). Permutational multivariate analysis of variance (PERMANOVA) was used to analyze differences in community structures. Based on Linear Discriminant Analysis (LDA), an LEfSe analysis plot was generated to analyze the unique signature microorganisms in different tissues of *N. candida*. The relative abundance of Amplicon Sequence Variants (ASVs) was compared using the R package (version 0.5.0) DESeq 2. Only *p* < 0.05 (FDR-adjusted) and log2 (fold change) > 2 or <−2 were considered significant differences in ASV.

## 3. Results

### 3.1. Fungal Contamination in Tissue Culture of N. candida

**Figure 1:**
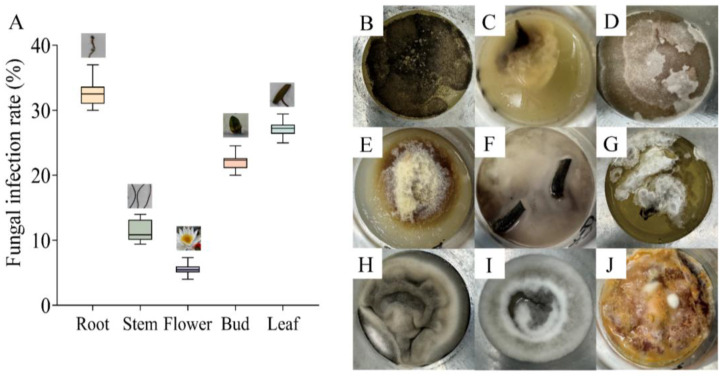
The infection rates (**A**) and growth morphology (**B**–**J**) of fungal phenotypes in the tissue culture bottles of root (**B**–**E**), leaf (**F**–**I**), stem (**F**–**I**), and bud (**J**) tissues of *N. candida* Presl.

Fungal contamination was observed in tissue culture of *N. candida*, with the highest infection rate of 33.33 ± 0.35% in the roots and 27.77 ± 0.35% in the leaves (Figure 1A). By contrast, the flowers had the lowest fungal infection rate (5.55 ± 0.16%). There were four fungi in root tissue (Figure 1B–E), and two fungi were found in leaf and stem tissue (Figure 1F–I). For bud tissue, only one fungus was observed (Figure 1J). These fungi were in different forms.

### 3.2. Biodiversity and Community Composition of Endophytic Fungi in the Tissues of N. candida

**Figure 2:**
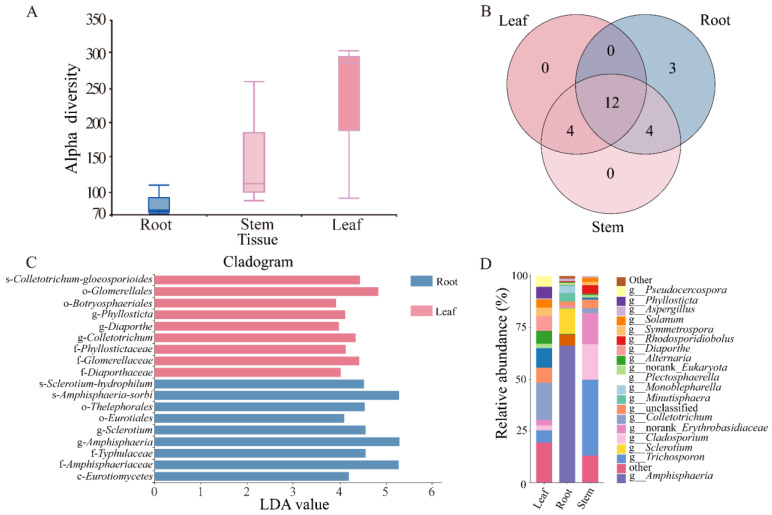
(**A**) Community composition of endophytic fungi in the root, stem, and leaf tissues of *N. candida* and the relative abundance of each species in different tissues. (**B**) Different colors represent different groups, in which the number of overlapping parts represents the number of species shared by multiple groups, and the number of non-overlapping parts represents the number of species unique to the corresponding group; (**C**) LEfSe analysis of endophytic fungal communities in different tissues of *N. candida*. Only the classification units that meet the linear discriminant analysis significance threshold > 2.0 are displayed and marked with color. (**D**) The horizontal axis is the number of each sample, and the vertical axis is the relative abundance ratio of the species.

There was no significant difference in the alpha and beta diversity of endophytic fungi in the roots, stems, and leaves of *N. candida* (Figure 2A, Tables S2 and S3). Compared to stem and leaf tissues, *Amphisphaeria*, *Sclerotium*, and *Minutisphaera* in the root tissue of *N. candida* exhibited higher abundances of 38.03%, 6.83%, and 2.41%, respectively. In stem tissue, *Trichosporonaceae*, norank-*Erythrobasidiaceae*, and *Rhodosporidiobolus* had high abundances of 6.43%, 2.65%, and 0.73%. In leaf tissue, *Colletotrichum* and *Plectosphaerella* had high abundances of 2.87% and 1.50% (Figure 2D). Notably, the root endophytic fungal community displayed greater richness and contained three unique fungal taxa––*Amanita*, *Kohlmeyeriopsis*, and *Monoblepharella*––which were absent from both stem and leaf tissues (Figure 2B).

LEfSe analysis showed that the dominant fungal species in the root tissue was the genus s-*Amphisphaeria*-*sorbi* within the family f-*Amphisphaeriaceae*, and the dominant fungus in the leaf tissue was of the order o-*Glomerellales* (Figure 2C). In the root tissue of *N. candida*, the pathogenic endophytic fungi *Sclerotium* had the highest abundance of 6.83% (Figure 2D). Compared to the stem and leaf tissues, the root tissue exhibited higher abundance of the pathogenic endophytic fungi *Aspergillus*, *Alternaria*, and *Cladosporium*, with relative abundances of 0.68%, 0.15%, and 0.40%, respectively (Figure 2D).

### 3.3. Identification of Endophytic Fungi in N. candida

**Figure 3:**
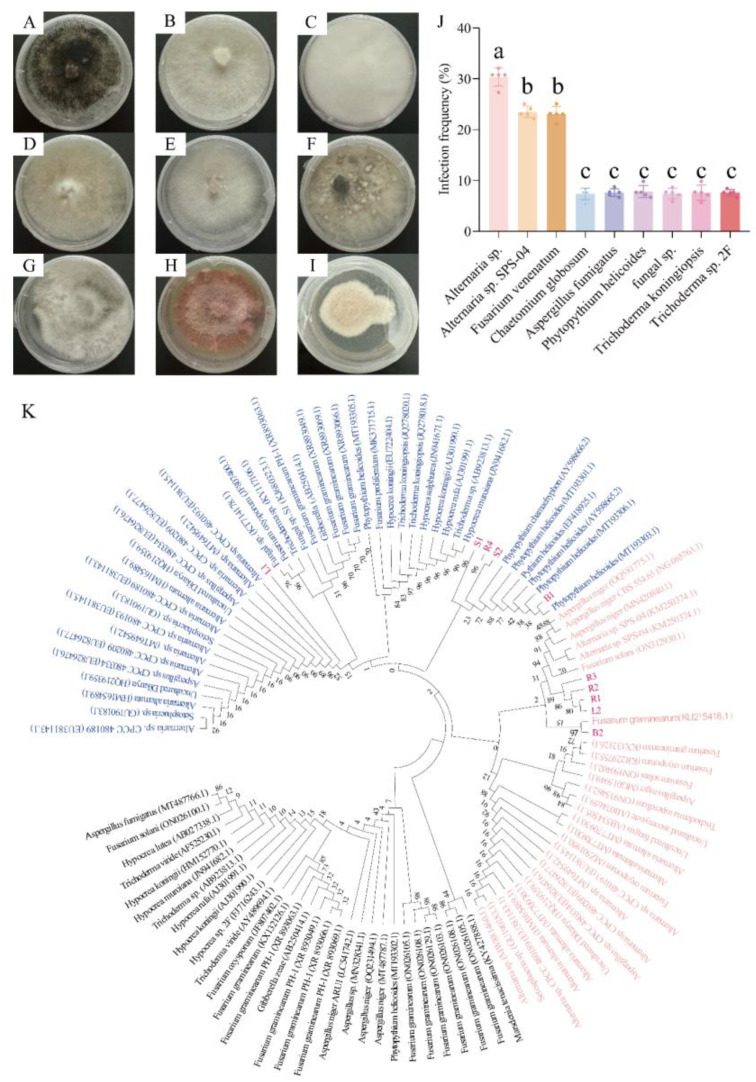
Endophytic fungi in the tissues of *N. candida*. (**A**–**D**) show fungi R1, R2, R3, and R4 isolated from the root tissue, (**E**,**F**) show fungi L1 and L2 isolated from the leaves, (**G**,**H**) show fungi B1 and B2 isolated from the buds, and (**I**) shows fungus S1 isolated from stem tissue culture of *N. candida*. (**J**) shows the infection rates of each endophytic fungi, and (**K**) shows the phylogenetic tree of the identified endophytic fungi. The branching pattern was generated by the adjacency connection method, and 1000 repeated bootstrap analyses were performed with 0.5 or 1.0 nucleotide substitutions per site. Note: Different letters in the figure indicate significant differences among the results (*p* < 0.05).

Nine endophytic fungi with different forms were isolated from the contaminated *N. candida* tissues (Figure 3A–I). The results of ITS sequencing showed that nine fungi were *A.* sp., *Chaetomium globosum*, *A.* sp. SPS-04, *Aspergillus fumigatus*, *P. helicoides*, *fungal* sp., *Trichoderma koningiopsis*, *F. venenatum*, and *T.* sp. 2F (Figure 3K, Tables S4 and S5). Among the isolated endophytic fungi, three pathogenic fungi––*A.* sp., *P. helicoides*, and *F. venenatum*––showed very high frequencies of occurrence of 30.77%, 23.08%, and 23.08%, respectively, in tissue culture (Figure 3J).

### 3.4. The Inhibition Effects of Five Drugs on Pathogenic Endophytic Fungi

**Figure 4:**
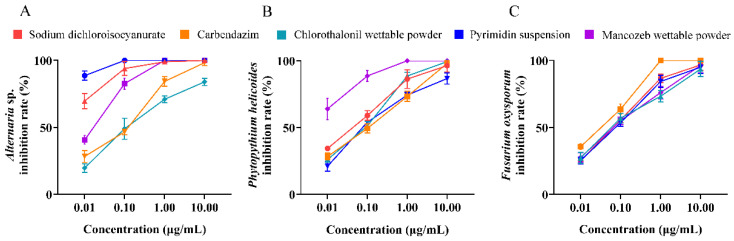
The inhibition effects of carbendazim, sodium dichloroisocyanurate, chlorothalonil wettable powder, pyrimidin suspension, and mancozeb wettable powder at different concentrations on three endophytic fungi: *Alternaria* sp. (**A**), *Phytopythium helicoides* (**B**), and *Fusarium venenatum* (**C**).

Five drugs were used to inhibit three isolated pathogenic endophytic fungi, and the results showed that the inhibition rates increased with increasing drug concentrations. Specifically, the inhibition rate of pyrimidin suspension against *A.* sp. reached 100% at 0.1 μg/mL (Figure 4A), and the inhibition rate of mancozeb wettable powder against *P. helicoides* reached 100% at 1 μg/mL (Figure 4B). Similarly, the inhibition rate of carbendazim against *F. venenatum* reached 100 % at 1 μg/mL (Figure 4C).

### 3.5. Application of Fungicide Combination in Tissue Culture

**Figure 5:**
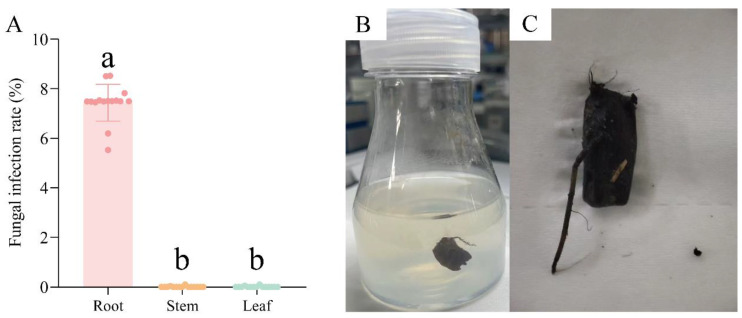
The infection rates of fungi in the root, stem, and leaf tissues (**A**) after the addition of fungicide combination and their effects on the redifferentiation of root tissue and callus to form organs during tissue culture of *N. candida* (**B**,**C**). Note: Different letters in the figure indicate significant differences among the results (*p* < 0.05).

Based on the results of the antifungal tests, 0.1 μg/mL pyrimidin suspension, 1 μg/mL mancozeb wettable powder, and 1 μg/mL carbendazim were jointly used in tissue culture of *N. candida*. After adding the above fungicide combination, the infection rate of root tissue was 7.5 ± 0.03%, and the infection rates of stem and leaf tissues decreased to 0% (Figure 5A). Moreover, tissue explants of *N. candida* grew normally after adding the fungicide combination (Figure 5B,C).

## 4. Discussion

This study demonstrated that proliferation of pathogenic endophytic fungi, including *A.* sp., *P. helicoides*, and *F. venenatum,* led to failure of tissue culture of *N. candida*, and a fungicide combination consisting of 0.1 μg/mL pyrimidin suspension, 1 μg/mL mancozeb wettable powder, and 1 μg/mL carbendazim could effectively reduce infection with pathogenic fungi and significantly enhance the success rate of tissue culture of *N. candida*.

### 4.1. Pathogenic Endophytic Fungi in N. candida Tissues

In this study, various endophytic fungal infections were observed during tissue culture of *N. candida*, and the roots exhibited the highest infection rate (33.33%). This finding was consistent with previous studies on tissue culture of bulbous plant species, including *Narcissus pseudonarcissus*, *Lilium humboldtii*, and *Tulipa* spp., in which subterranean tissues had an elevated risk of microbial infection [29,30,31]. Moreover, the three endophytic fungi *A.* sp., *P. helicoides*, and *F. venenatum* showed relatively high frequencies in tissue culture of *N. candida*, especially in the root tissue. These fungi are well-known plant pathogens associated with tissue culture failures and involved in diverse pathologies, including brown spot, root rot, and Fusarium wilt [20,24,29,32,33,34,35]. High infection rates (20–30%) of *A.* sp. and *F. venenatum* have been observed in *Musa* spp. and *Chlorophytum borivilianum* micropropagation systems [23,36]. High-throughput sequencing of root, stem, and leaf tissues was further conducted to systematically characterize endophytic fungal communities. Root tissues harbored pathogenic genera, including *Amphisphaeria*, *Sclerotium*, and *Minutisphaera*, which could induce stem rot and Fusarium wilt in solanaceous crops (*Solanum lycopersicum*, *Capsicum annuum*) and sunflower (*Helianthus annuus*) [37]. *Trichosporonaceae*, norank-*Erythrobasidiaceae,* and *Rhodosporidiobolus* accounted for a relatively high proportion in the stem tissue of *N. candida*, while *Colletotrichum*, *Plectosphaerella*, and *Diaporthe* accounted for a relatively high proportion in the leaf tissue. These endophytic fungi have not been reported in plant pathogenicity. Quantitative analysis showed that the roots contained 8.06% pathogenic fungal species by abundance, substantially exceeding the stems (3.26%) and leaves (4.29%). Thus, these pathogenic endophytic fungi might be the main reason for failure of tissue culture of *N. candida*.

### 4.2. Development and Validation of an Antifungal Formulation for Tissue Culture of N. candida

Considering that suppression of endophytic pathogens might help enhance the success rate of tissue culture, five systemic fungicides were selected to conduct inhibition experiments [38,39,40,41,42]. Among them, carbendazim and pyrimidin suspension, sodium dichloroisocyanurate, and chlorothalonil wettable powder had good control effects on *Fusarium* and *Pythium capsici* [39,40,42,43]. These antifungal drugs at different concentrations were added into MS medium, and the results showed that 0.1 μg/mL pyrimidin suspension and 1 μg/mL sodium dichloroisocyanurate could completely inhibit the growth of *A.* sp., *P. helicoides*, and *F. venenatum*, respectively. The inhibition rate of *P. helicoides* reached 100% after the addition of 1 μg/mL mancozeb wettable powder or 10 μg/mL chlorothalonil wettable powder. Previous studies reported that 6 μg/mL carbendazim could completely inhibit growth of *A.* sp. in tissue culture of sugarcane (*Saccharum officinarum* L.) [44], and growth of *P. helicoides* was completely inhibited by adding 8 μg/mL ofloxacin during tissue culture of *Hygrophila polysperma* [19]. Similarly, the best inhibitory effect on *F. venenatum* was 1 μg/mL carbendazim and 10 μg/mL chlorothalonil wettable powder in this study. It was reported that the addition of 9 μg/mL gatifloxacin could completely inhibit the growth of *F. venenatum* [45]. By contrast, this study used a lower dose of drugs to inhibit endophytic pathogens and developed a formulation containing 1 μg/mL carbendazim, 1 μg/mL mancozeb wettable powder, and 0.1 μg/mL pyrimidin suspension in a 1:1:1 volumetric ratio (*v*/*v*/*v*) to prevent fungal infection during tissue culture of *N. candida.*

To validate the usefulness of the antifungal formulation, we supplemented the medium with the antifungal cocktail during *N. candida* tissue culture. The infection rate of roots decreased to 7.5%, and the infection rate of stems and leaves decreased to 0%. Moreover, there was no adverse effect on the growth of *N. candida* explants. Studies have shown that the addition of a mixture of 4% gatifloxacin, 1% carbendazim, and 25 mg/L piperacillin and levofloxacin could reduce the infection rates of *Chlorophytum borivilianum* and banana tissue culture to 10% and 6%, respectively [23,36]. However, high doses of these drugs led to browning and albino symptoms in explants. The addition of a mixture of 10 μg/mL kanamycin and 5 μg/mL chloramphenicol could completely inhibit fungal infections in tissue culture of the aquatic plant *Hygrophila polysperma*, but a low dose of the drug combination led to serious contamination of the explants [19]. By contrast, the formulation prepared in this study had a lower dose of drugs and a better effect in tissue culture. On the other hand, the source of explants is one of the key factors affecting the success of tissue culture [30]. Because root tissues exhibited higher abundances of pathogenic endophytic fungi, we suggest that the use of stems and leaves with relatively few endophytic fungi could effectively increase the success rate of *N. candida* tissue culture.

## 5. Conclusions

An antifungal cocktail (0.1 μg/mL pyrimidin suspension, 1 μg/mL mancozeb wettable powder, and 1 μg/mL carbendazim) offered an effective solution to control endophytic fungal contamination in *N. candida* tissue culture. Beyond its usefulness in the protection of the germplasm resources and the propagation of *N. candida*, it also lowers restoration costs for other endangered aquatic species threatened by microbial infection. The findings of this study pave a viable way for large-scale ecological and commercial applications of valuable and rare plants.

## Data Availability

The raw data supporting the conclusions of this article will be made available by the authors on request.

## Supplementary Materials

The following supporting information can be downloaded at: https://www.mdpi.com/article/10.3390/microorganisms13051103/s1. Table S1: Screening of antifungal drugs, alpha and beta diversity indices of endophytic fungi in the tissues of *N. candida*; Table S2: Alpha diversity index table; Table S3: Beta diversity index table, ITS sequencing results, and morphological characteristics of endophytic fungi isolated from water lilies; Table S4: BLAST sequence alignment result and morphological characteristics; Table S5: Morphological characteristics of nine endophytic pathogenic fungi.
