# Supplementary material for: Isolation, Identification, and Control of Pathogenic Endophytic Fungi in Nymphaea candida Presl Tissue Culture
Source: Microorganisms. 2025 May 10;13(5):1103. doi: 10.3390/microorganisms13051103 (PMC12114180; doi:10.3390/microorganisms13051103)
Supplement: Supplementary file 1 [file microorganisms-13-01103-s001.zip › microorganisms-3600363-supplementary.pdf]

Isolation, identification and control of pathogenic endophytic fungi in tissue culture of *Nymphaea candida* Presl

Yuwei Xing[1,#], Cong Liu[1,#], Xumeng Cui[1], Haonan Lv[1], Jun Wang[1,*]

[1]Colleges of Marine Life Sciences, Ocean University of China, 5 Yushan Road, Qingdao, 266003, Shandong Province, China.

#Y. Xing and C. Liu contributed equally to this work.

*Corresponding author

Phone: +86-532-82031962

Fax: +86-532-82031962

Email: wangjun@ouc.edu.cn

Screening of antifungal drugs

The antifungal effects of five antifungal drugs in different plant tissue culture processes (Table S1).

Table S1 Screening of antifungal drugs

Fungicide	Concentration Range	Primary Target Fungal Species	Antifungal Mechanism	Plant Species
Sodium dichloroisocyanurate	0.5-2.0 mg/L	*Pythium* spp., *Fusarium oxysporum*	Releases active chlorine to oxidize cellular components	*Musa* spp., *Dendrobium* spp.
Carbendazim	5-10 μg/mL	*Penicillium digitatum*, *Aspergillus niger*	Inhibits β-tubulin polymerization	*Saccharum officinarum*, *Solanum tuberosum*
Chlorothalonil	3-8 μg/mL	*Alternaria alternata*, *Botrytis cinerea*	Blocks mitochondrial electron transport chain	*Nicotiana tabacum*, *Solanum lycopersicum*
Pyrimidin suspension	0.05-0.2 μg/mL	*Magnaporthe oryzae*, *Podosphaera leucotricha*	Inhibits melanin synthesis and sterol metabolism	*Oryza sativa*, *Malus domestica*
Mancozeb	1-3 μg/mL	*Phytophthora infestans*, *Rhizoctonia solani*	Chelates metal cofactors and disrupts membrane structure	*Vitis vinifera*, *Solanum tuberosum*

Alpha and Beta diversity index of endophytic fungi in the tissues of *N. candida*

This study measured the alpha diversity index and beta diversity index of endophytic fungi in the roots, stems, and leaves of *Nymphaea candida* Presl, and the results indicated no significant differences (Table S2 and S3).

Table S2 Alpha diversity index table

Treatments	ACE	Chao	Shannon	Simpson	Coverage	Sobs
Root	0.37±0.09	90.53±28.36	79.00±19.16	0.37±0.17	1.60±0.41	127557.70±1703.95
Stem	0.32±0.07	157.83±85.01	144.67±88.33	0.33±0.08	1.54±0.25	123420.00±3203.65
Leaf	0.34±0.05	229.81±115.14	278.33±12.42	0.32±0.01	1.78±0.08	113099.70±8903.84

Table S3 Beta diversity index table

Treatments	PCOA1	PCOA2	PCOA3	PCOA4	PCOA5	PCOA6
Root	‘- 0.33±0.45	’-0.00±0.04	0.03±0.08	0.03±0.07	‘- 0.00±0.01	’-0.01±0.01
Stem	0.16±0.03	’-0.04±0.18	‘-0.03±0.11	’-0.00±0.03	0.04±0.02	0.01±0.04
Leaf	0.17±0.00	0.04±0.03	‘-0.00±0.03	’-0.03±0.02	‘0.03±0.02	’-0.00±0.03

ITS sequencing results and Morphological characteristics of endophytic fungi isolated from water lilies

Nine endophytic pathogenic fungi were isolated and identified, specifically: *Alternaria* sp., *Chaetomium globosum*, *Alternaria* sp. SPS-04, *Aspergillus fumigatus*, *Phytopythium helicoides*, *fungal* sp., *Trichoderma koningiopsis*, *Fusarium venenatum*, and *Trichoderma* sp. 2F (Table S4). Morphological characteristics of nine endophytic pathogenic fungi (Table S5).

Table S4 Blast sequence alignment result and morphological characteristics

Number	Fungi	Classify	Similarity	Approximate strain entry number
L1	*Alternaria* sp.	Ascomycotina	99.92%	MT649542.
R1	*Chaetomium globosum*	Ascomycetes	99.77%	JQ964323.1
L2	*Alternaria* sp. SPS-04	Ascomycotina	99.55%	KM250374.1
R2	*Aspergillus fumigatus*	Hemiomycetes	99.55%	MT487766.1
B1	*Phytopythium helicoides*	Anisomastigota	99.78%	AY598665.2
R3	*fungal* sp.	Eumycophyta	99.70%	KT714178.1
R4	*Trichoderma koningiopsis*	Hemiomycetes	99.61%	JQ278020.1
S1	*Fusarium venenatum*	Ascomycotina	99.63%	LN649232.1
S2	*Trichoderma* sp. 2F	Hemiomycetes	99.85%	FJ716243.1

Table S5 Morphological characteristics of nine endophytic pathogenic fungi

Number	Colour	Surface evenness	Edge evenness	Depth of mycelium	Is there any exudate on the surface
L1	Offwhite	Unevennes	Relatively regular	Relatively deep, closely combined with the culture medium	Yes
R1	Offwhite	Unevennes	Irregular	relatively shallow, loosely with the culture medium	Yes
L2	Pink	Relatively flat	Relatively regular	Relatively deep, closely combined with the culture medium	No
R2	Tawny	Unevennes	Relatively regular	Relatively deep, closely combined with the culture medium	No
B1	Offwhite	Unevennes	Relatively regular	Relatively deep, closely combined with the culture medium	No
R3	Offwhite	Relatively flat	Relatively regular	Relatively deep, closely combined with the culture medium	Yes
R4	Offwhite	Relatively flat	Relatively regular	Relatively deep, closely combined	Yes

				with the culture medium	
S1	Pink	Unevennes	Irregular	Relatively deep, closely combined with the culture medium	No
S2	Offwhite	Unevennes	Relatively regular	Relatively deep, closely combined with the culture medium	No
